# Optimising remote site airway management kit dump using the SCRAM bag—a randomised controlled trial

**DOI:** 10.1186/s13741-020-00140-w

**Published:** 2020-04-14

**Authors:** Barry M. Schyma, Andrew E. Wood, Saranga Sothisrihari, Paul Swinton

**Affiliations:** 1 Trauma Anaesthesia Group, Department of Anaesthesia, Royal London Hospital, London, UK; 2 Paramedic, ScotSTAR, Scottish Ambulance Service, Paisley, UK

**Keywords:** Airway, Human factors, Kit dump, Remote site

## Abstract

**Background:**

Emergency airway management may be required at any hospital location. Remote site management is associated with increased airway morbidity and mortality. Poor planning and interrupted workflow are significant contributors. Equipment may be unfamiliar, difficult to locate or inadequate.

The SCRAM (Structured CRitical Airway Management) bag aims to provide a portable, structured and reproducible approach to airway management preparation. We hypothesised that SCRAM bag use reduces equipment preparation time, the rate of error and operator cognitive load.

**Methods:**

Fifty experienced anaesthetists were randomised into two groups and asked to prepare (kit dump) for and manage a simulated remote site difficult airway scenario. The control group (*n* = 25) used a standard resuscitation trolley while the experimental group used the SCRAM bag (*n* = 25). The primary outcome was time taken to kit dump completion (seconds). Secondary outcomes were the number of errors and self-reported difficulty (100 mm visual analogue scale).

**Results:**

Using the SCRAM bag, a 29% reduction in kit dump time (111.7 ± 29.5 vs 156.7 ± 45.1, *p* = 0.0001) was noted. Participants using the SCRAM bag reported it to be less challenging to use (18.36 ± 16.4 mm vs 50.64 ± 22.9 mm, *p* < 0.001), and significantly fewer errors were noted (1 (IQR 1–3) vs 8 (IQR 5–9), *p* = 0.03) (87.5% reduction in the total number of errors).

**Conclusion:**

The SCRAM bag facilitates a quicker, less challenging kit dump with significantly fewer errors. We propose that this would reduce delay to airway management, reduce cognitive load and provide an improved capability to manage anticipated and unanticipated airway events.

Airway management occurring outside the operating theatre is associated with increased morbidity and mortality (Walz et al., 2007; Cook et al., 2011a; Cook et al., 2011b; Cook et al., 2012). Both the emergency departments (Carlson & Wang, 2013; Kerslake et al., 2015; Nakao et al., 2015) and critical care units (Walz et al., 2007; Jaber et al., 2006; Simpson et al., 2012) have been highlighted as areas of increased risk; however, an anaesthetist may be asked to manage a threatened airway at any location within the hospital. Brindley et al. (Brindley et al., 2017) describe beautifully how working in these ‘remote’ or ‘satellite’ environments ‘requires concurrent mastery of anatomically, physiologically, and situationally difficult airways’. Causes are multifactorial (Rosenstock et al., 2006; Green, 2009; Schnittker et al., 2018). Contributors include operator skill and experience, environmental issues, the availability of skilled assistance and inadequate planning. Equipment may be difficult to locate, unfamiliar or inadequate. These factors may lead to delayed airway management and blunt an operator’s ability to perform in a stressful scenario.

Improving workflow improves performance. The SCRAM (Structured CRitical Airway Management, Fig. 1) bag was initially designed for pre-hospital use and has an optimal ergonomic layout that includes a ‘shadow template’ cognitive aid. It is designed to provide a portable, structured and reproducible approach to preparing both drugs and equipment for advanced airway management (Swinton et al., 2018).

**Fig. 1:**
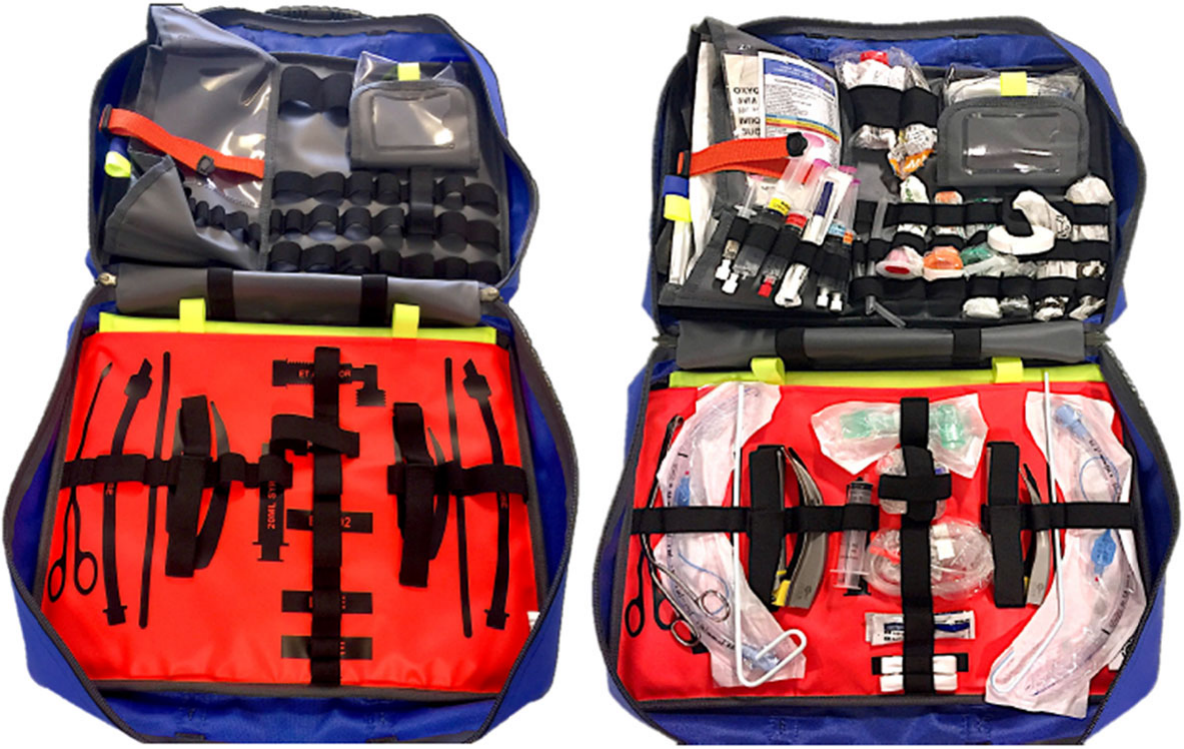
The Adult SCRAM (Structured CRitical Airway Management) Bag is an Emergency Airway Bag which provides a structured reproduceable approach to airway management. The ‘shadow template’ acts as a cognitive aid to facilitate kit dump

Many airway challenges experienced in the pre-hospital arena are similar to those experienced in remote hospital sites. Swinton et al. (Swinton et al., 2018) investigated SCRAM bag use in experienced paramedics and demonstrated a significant reduction in drug, but not equipment, preparation time in addition to a reduction in the error rate in both phases. Pre-hospital providers are very familiar with rapid equipment preparation in the field. Anaesthetists, especially those who predominantly work in the theatre environment, are not and the SCRAM bag design may be of benefit.

We hypothesised that the use of the SCRAM bag by experienced anaesthetists could facilitate remote site airway management planning by reducing the time taken to prepare equipment, the error rate and operator cognitive load.

## Methods

This simulation-based study was deemed exempt from formal review by the local institutional review board (IRAD ID: 224359). Written informed consent was obtained from all participants.

Participants were recruited from the Anaesthetic Department at a busy major trauma centre. All participants had a minimum of 2 years’ experience in anaesthesia and emergency airway management and were familiar with the Difficult Airway Society guidelines (Frerk et al., 2015) for the management of an unexpected difficult airway.

Participants were blinded to the study outcomes. They were asked to manage a simulated remote site difficult airway scenario using an airway training mannequin. They were asked to prepare the equipment required (kit dump) in keeping with the Difficult Airway Society guidelines (Frerk et al., 2015) including equipment for front of neck access. Participants were told that they must declare when they had all required equipment ready before they could proceed to managing the airway. The scenario was stopped on completion of the kit dump.

We felt, for a number of governance reasons, that the majority of hospitals would not stock a remote site airway bag with induction drugs, especially those on the controlled drugs register. While the SCRAM bag has capacity for drug storage, we elected to only test the equipment preparation component.

Participants were block randomised by blindly selecting a letter from a bag. The letter allocation specified one of two groups:

- The control group (‘trolley’) were provided with a fully stocked National Health Service (NHS) resuscitation trolley and asked to extract the equipment required. Resuscitation trolleys are found throughout all hospitals (Bowden & Smith, 2017; Gallacher, 2017) in the UK. Most institutions worldwide will have some form of similar compact storage for equipment to facilitate advanced life support. The resuscitation trolley is frequently the source of airway management equipment in remote sites where difficult airway trolleys have not been placed. To ensure fidelity, all non-airway equipment that would also be stocked on a resuscitation trolley was included. The layout was standardised as per hospital protocol. A standardised resuscitation trolley does not contain the ‘shadow template’ cognitive aid.
- The ‘SCRAM’ group were provided with a fully stocked SCRAM bag (Fig. 1).

The time taken for kit dump was recorded and performance monitored by a study investigator. Participants were asked to rate their perceived degree of difficulty with the trolley or SCRAM bag using a 100 mm visual analogue scale. Upon conclusion, the kit dump was inspected for omission errors using a standardised checklist. Table 1 details the equipment expected to be present and prepared for use for a kit dump to be considered complete. We expected laryngoscope bulbs and endotracheal tube pilot balloons to be checked, and failure to do this was recorded as an error.

**Table 1:** Kit dump checklist

• Nasopharyngeal airway (two sizes)	• 20 ml syringe
• Lubricating gel	• Tube tie
• Supraglottic device (two different sizes)	• Bougie/stylet
• Laryngoscope × 2 (two different sizes, must be checked)	• Scalpel
• Endotracheal tube (two different sizes, must be checked)	Magill forceps
• Catheter mount	

Participants were not allowed any familiarisation with the equipment prior to undertaking the scenario, although all participants will have been aware of the resuscitation trolley content during daily activity within the health trust.

The primary outcome was the time taken from the moment the participant touched the trolley or bag until completion of the kit dump (seconds). Completion was recorded as the time at which the operator declares that all equipment is available and they are ready to proceed with airway management. Secondary outcomes were preparation errors, recorded during and upon conclusion of the kit dump, and operator-perceived difficulty (100 mm visual analogue scale). Errors recorded included items of equipment not prepared (omissions) and items of equipment not checked.

Data derived from a previous unpublished pilot study undertaken by the Scottish Ambulance Service suggested a mean time of approximately 300 s to achieve an adequate kit dump using a standard airway bag and a 20% reduction using the SCRAM bag. We hypothesised that the time to prepare the kit using a resuscitation trolley would be similar to that of the SAS airway bag and the time to prepare the equipment using the SCRAM bag would be approximately 250 s. Forty-four participants were required to have a 90% chance of detecting a difference at the 5% significance level (power 0.8, alpha error 0.05). We intended to recruit a total of 50 participants to allow for non-compliance (https://www.sealedenvelope.com/power/continuous-superiority).

Statistical analysis was performed using Microsoft Excel. Parametric data are presented as mean ± standard deviation and analysed using student *T* tests. Non-parametric data is presented as median ± interquartile range and analysed using chi-squared testing. *p* values were set at < 0.05 for statistical significance.

## Results

Fifty participants were recruited and were included in the final analysis. Participant age and experience were similar between the two groups (Table 2).

**Table 2:** Table of results

	Trolley	SCRAM	
	*n* = 25	*n* = 25	
Age, years (IQR)	34 (32–38.5)	36.5 (32–41)	*p = 0.68*
Years of experience, years (IQR)	8 (5.5–12)	7.5 (3.5–14.5)	*p = 0.41*
Completion time (s)	157 ± 45	112 ± 30	*p = 0.0001*
Errors (*n*, (IQR)) - Total - Omission - Checking	8 (5–9) 5 (3–7) 3 (2–4)	1 (1–3) 1 (1–2) 0 (0–1)	*p = 0.03*
Perceived challenge (mm)	50.64 ± 22.9	18.36 ± 16.4	*p < 0.001*

Table 2 demonstrates that the time for kit dump completion was 29% lower using the SCRAM bag (112 ± 30 vs 157 ± 45, *p* = 0.0001, 95% CI ± 22.1) There was an 87.5% reduction (*p* = 0.03, 95% CI 1.67) in total errors using the SCRAM bag compared with the resuscitation trolley. Twenty vs 4% (*p* = 0.04) recorded an error free kit dump using SCRAM bag versus trolley. The majority of errors in each case were errors of omission. The principal items omitted were the backup laryngoscope, a second endotracheal tube and a bougie. The SCRAM bag was perceived as 68.3 % less difficult to use than the resuscitation trolley.

## Discussion

Planning and preparation of equipment are important components of emergency airway management and have been highlighted as areas for improvement in remote site airway management. Our data demonstrates that the SCRAM bag facilitates a quicker, less challenging kit dump with, perhaps more importantly, significantly fewer errors. We propose that this would reduce delays to remote site airway management, reduce cognitive load and provide an improved capability to manage anticipated and unanticipated airway events.

Managing a time critical, unexpected threatened airway in a potentially unfamiliar remote site is challenging. The patient may have significant respiratory or haemodynamic inadequacy and may not be fasted. The environment may be noisy and constrained with challenging ergonomics. The team may be unfamiliar and a trained assistant/dedicated support technician, such as an operating department practitioner, respiratory therapist or anaesthetic nurse, may not be immediately available to assist. The quality of clinical care delivered must remain the same, but the potential for delay and error is significantly greater.

Unfamiliarity with the location and use of emergency airway equipment in a hospital is a worldwide concern (Green, 2009; Schnittker et al., 2018). Emergency airway equipment in hospital locations outside the operating theatre is commonly co-located with other cardiorespiratory resuscitation equipment. Within the National Health Service, this is on a resuscitation trolley; however, similar setups are employed worldwide. The setup is not standardised between hospitals or healthcare systems but have similar features. They will be relatively mobile, compact and contain all the equipment necessary to support provision of the institutional advanced life support guidelines. Co-locating all resuscitation equipment allows portability but means that a large team must all obtain equipment from the same trolley at the same time. The compact nature of the trolley limits the scope for single system organisation. Difficult airway trolleys allow this (Difficult Airway Society, 2017) and are commonly found in operating theatres, critical care and the emergency department; however, the nature of the complex equipment they contain makes them less portable, and locating these in all clinical and non-clinical areas is impractical. The SCRAM bag is not intended to replace the difficult airway trolley; rather, it provides a portable solution for clinical areas where no trolley can be placed.

Many departments have undertaken the implementation of airway bags as a means of improving remote site airway management (Wijesuriya & Brand, 2014). These are lightweight and can be brought from a central store to the remote site. While this solves the problem of portability, most focus on storage and transportation of equipment rather than equipment preparation, process improvement and performance enhancement. The result may be a disorganised selection of multiple pieces of equipment. Hick’s law (Hick, 1952) states that the time taken to make a decision increases logarithmically with the number of options presented. An overstocked, disorganised airway bag is not the optimal arrangement.

Standardising the kit dump process and the use of cognitive aids reduces the number of decisions an operator must make, ensures equipment availability and has been recommended as a strategy for reducing error (Schnittker et al., 2018; Benger & Hopkinson, 2011; Marshall, 2013; Lockey et al., 2014; Long et al., 2016; Chrimes, 2016). We observed that those using the SCRAM bag performed a kit dump significantly quicker. Reducing kit dump time reduces the delay in securing an airway. The SCRAM bag is currently being used in both pre-hospital and hospital environments in the UK, Australia and other parts of the world. It provides a structured, reproducible approach to airway management. By standardising and optimally organising equipment in a manner that reflects the series and sequence of steps required for the intervention, it becomes a useful “tool” for cognitive load reduction. We believe that this reduces the number of decisions that must be made which leads to a significant reduction in set up time and error rate. It is interesting that the reduction in equipment kit dump time that we have observed in experienced anaesthetists was not observed when the SCRAM bag was used by paramedics (Swinton et al., 2018). This may reflect the differing controls that have been used between the two studies but may also represent a training gap in equipment preparation in anaesthetists compared with kit dump providers. Anaesthetists are fortunate to have the skilled assistance of operating department practitioners, respiratory therapists and anaesthetic nurses. This luxury may have the effect of becoming under skilled in equipment setup and highlight the importance of aids when skilled assistance is unavailable.

It is difficult to know whether the 45-s reduction in kit dump time is directly clinically relevant in the majority of scenarios, although the SCRAM bag is clearly a beneficial intervention in any ‘marginal gains’ strategy. We would suggest that the reduction in kit dump time observed may be an underestimation. Our control study simulation used a single operator to extract equipment from the resuscitation trolley. In reality, there is competition for access to extract equipment and our control time may be artificially fast. In an emergency scenario, other team members will require items such as drugs, intravenous cannulas and fluids from other drawers at the same time as airway management is planned.

Performing an intervention more quickly must not compromise safety and quality. Human error is of particular interest when considering patient safety (Stelfox et al., 2006; Cook et al., 2010; Keebler et al., 2018), and we must design systems to mitigate it. A high rate of error, especially omission error, was seen in the control group. This was seen despite the participant’s previous familiarity with the resuscitation trolley. The most frequent omission was a bougie, a backup laryngoscope and an alternative endotracheal tube size. These omissions occurred despite a calm and ordered simulated scenario and may be amplified in a ‘real life’ scenario. The impact of omissions on a hypoxic patient with a potential difficult airway is obvious. This may be a bigger problem in a remote site where the nearest store is a significant distance away, rather than in a theatre cupboard in close proximity.

The SCRAM bag reduced the total number of kit dump errors by 87.5%. A greater number of participants were able to demonstrate an error free kit dump, and those making errors made far fewer. This secondary outcome may be of greater impact clinically than our initial primary outcome. The ‘shadow template’ cognitive aid visually prompts the user to ensure all the necessary equipment is prepared in advance. Other studies using airway templates have seen similar reductions in omission rate (Long et al., 2016). It may be that the presence of the shadow template is what gives the SCRAM bag the biggest advantage over the resuscitation trolley where it is absent. The very few omission errors witnessed using the SCRAM bag were reported by operators as conscious decisions not to select a piece of equipment. Anecdotally, this was seen in the more experienced operator. This observation reminds us that standardisation of a process also requires operator compliance and that its existence does not necessarily mean that it will be used in a clinical scenario (Marshall & Mehra, 2014). Non-compliance with Difficult Airway Society guidelines has been previously reported (Green, 2009). We found that the equipment appeared to be checked more thoroughly using the SCRAM bag, perhaps owing to reduced cognitive load and increased operator bandwidth availability. The significant reduction in operator-perceived kit dump difficulty may also support this.

This simulation-based study has a number of limitations. We used our institutional resuscitation trolley as a control. This is comparable to most other resuscitation trolleys in the National Health Service but may not be comparable to systems used in other non-UK institutions. The study was designed to recreate an actual remote site airway emergency; however, we were unable to fully recreate all the stressors the operator would be experiencing in this situation. We cannot discount the Hawthorne effect (Gale, 2004), and the study may not absolutely reflect how an operator may behave in an actual scenario. If this scenario were to play out in a non-simulated environment, we feel that the preparation time and error rate in both control and experimental groups is likely to increase; however, we feel that the effect seen in the experimental group would be preserved. This warrants further investigation. This study does not directly demonstrate that the SCRAM bag reduces remote site airway morbidity and mortality or improve direct patient outcomes; however, its inclusion within a remote site airway management bundle is appealing.

## Conclusion

The SCRAM bag reduces kit dump error and improves speed in remote site airway management. It may reduce the time to definitive airway management. Further work is required, however, to ensure that it has an impact on patient airway morbidity and mortality.

## Data Availability

Please contact author for data requests. The datasets generated and/or analysed during the current study are not publicly available as we have no platform for publication but are available from the corresponding author on reasonable request.

## Abbreviations

CI: Confidence interval
NHS: National Health Service
SAS: Scottish Ambulance Service
SCRAM: Structured Critical Airway Management
